# Orientation-and polarization-dependent optical properties of the single Ag nanowire/glass substrate system excited by the evanescent wave

**DOI:** 10.1038/srep25633

**Published:** 2016-05-09

**Authors:** Mu Yang, Wei Cai, Yingjie Wang, Mengtao Sun, Guangyi Shang

**Affiliations:** 1 Department of Applied Physics, Laboratory of Micro-nano Measurement-manipulation and Physics (Ministry of Education), Beihang University, Beijing, 100191, People’s Republic of China; 2 Beijing National Laboratory for Condensed Matter Physics, Beijing Key Laboratory for Nanomaterials and Nanodevices, Institute of Physics, Chinese Academy of Science, Beijing, 100190, People’s Republic of China

## Abstract

As an important plasmon one-dimensional material, orientation- and polarization-dependent properties of single Ag nanowires/glass substrate system are investigated by a powerful platform consisting of evanescent wave excitation, near-/far-field detection and a micromanipulator. In the case of the nanowire perpendicular or parallel to the incident plane and *p*- or*s*-polarized evanescent excitation respectively, optical properties of the nanowire is measured both in far-field and near-field. For the perpendicular situation, scattering light from the nanowire shows strong dependence on the polarization of incident light, and period patterns along the nanowire are observed both in the near- and far-field. The chain of dipole model is used to explain the origin of this pattern. The discrepancy of the period patterns observed in the near- and far-field is due to the different resolution of the near- and far-field detection. For the parallel case, light intensity from the output end also depends on the incident polarization. Both experimental and calculation results show that the polarization dependence effect results from the surface plasmon excitation. These results on the orientation- and polarization-dependent properties of the Ag nanowires detected by the combination of near- and far-field methods would be helpful to understand interactions of one-dimensional plasmonic nanostructures with light.

The combination of optics and nanotechnology reveals a new way to enter the nano optical world^1^,^2^,^3^,^4^,^5^. Based on the effects of surface plasmon polaritons (SPPs) and localized surface plasmons (LSPs),optical devices can be reduced from microns to nanometer scale^6^,^7^,^8^,^9^. Such plasmonic devices are able to confine light energy into nanoscale with high spatial localization and the density of state^10^,^11^,^12^,^13^,^14^, which have not been done so well by other systems until now^15^. Among a variety of plasmonic elements, one-dimensional nanowires have attracted great interest due to their unique properties and wide potential applications in nano antennas^16^,^17^,^18^, sub-wavelength waveguides^19^,^20^,^21^,^22^,^23^, and high-resolution imaging^24^,^25^. It has been noted that these properties and applications are closely related to the polarization of light and a lot of related work has been reported recently. It was found that dipole and high-order surface plasmon resonant modes along the single nanowire can be tuned by changing the incident polarization^26^. Different SPP propagation modes were demonstrated in the case of different detection polarizations when it is excited on the nanowire^27^,^28^. Ag nanowire array grown on the grating shows distinguishing diffraction spectra with different polarized illumination light, showing potential applications in refractive index measurement and bio-sensing^29^.

For most experimental studies, nanowires under the investigation are generally prepared by using chemically synthesized methods, which are then deposited onto a substrate. Such samples consist of a large number of nanowires with arbitrary orientations. In this case, it is unable to detect optical properties of a single nanowire when they interact with light at the nanometer scale. In addition, the far-filed arrangements for the nanowire excitation are widely used. Sanders *et al*. demonstrated that the single Ag nanowire can guide and fan out light when it is excited at one end by the far-field method^30^. Miljkovic *et al*. studied the near-field and far-field properties of the Ag nanowire theoretically and their results are in agreement with the experimental noes well^31^. In the published papers above, the far-field excitation at the one end of nanowires are mostly realized with an objective lens. In the case, the scattering light from the excitation spot may interference with the collecting light^32^. Furthermore, the scattering properties by the whole nanowire are unable to be explored by the objective lens-based excitation method due to the strong background scattering light and weak signal from the single nanowires. Fortunately, an evanescent wave generated by the fiber taper or internal total reflection is an effective way for excitation without the strong background. For the fiber taper, light scattered from the distal end of a fiber can provide a broad distribution of wave vector, including the evanescent ones, which offer the needed wave vectors to match the momentum of photons and SPPs. Recently Gu *et al*. excited the Ag and Au nanowires evanescently by the fiber taper with a high technical operation and highly efficient coupling of light from fiber taper to nanowire has been obtained^33^. For internal total reflection, when the incident angle of light is larger than the critical angle (θ_c_ = sin^−1^(1/n_glass_)), nanowires placed on the surface can be excited by the evanescent wave. In fact, both the fiber taper and the internal total reflection provide a background-free excitation. Although the fiber taper configuration has the advantage of a high integrated level and miniaturized size, the internal total reflection are used in our present experimental setup for easy combination with a SNOM to image with a high resolution and with a micromanipulator to control the position and orientation of the single nanowire. Obviously, such the experimental setup combined with evanescent wave excitation, near-/far-field detection and a micromanipulator is a more appropriate choice for the investigation of the single nanowire.

Herein, we present orientation- and polarization-dependent properties of a single Ag nanowire measured by means of the combined experimental setup. The Ag nanowires are produced by a wet chemical method and a selected single nanowire is placed on a glass substrate using the micromanipulator (MMO-202ND, Narishige). When the nanowire orients perpendicular to the incident plane of light, the optical intensity due to scattering of the nanowire is detected by far-and near-field optical techniques^34^,^35^ as it is excited by the *p*-or *s*-polarized evanescent wave produced by the Dove prism. Periodic patterns along the nanowire axis are observed both in the far- and near-field regions. A chain of dipole oscillation model is used to explain the origin of the patterns. As the nanowire is parallel to the incident plane, the emitting light intensity from the distal ends of the nanowire as a function of the polarization state of incident light is measured. Intensity distribution obtained in the near-field region is analyzed both experimentally and theoretically. The work demonstrates that the experimental setup with multi-functionality of the micro manipulating for orientation controlling, the Dove prism-based evanescent wave excitation and far-/near-field detection, is an effective means to investigate properties of the single nanowire. The obtained results would be helpful to a deeper understanding of nanowire properties for applications in nanoscale optical devices.

## Results

**Figure 1:**
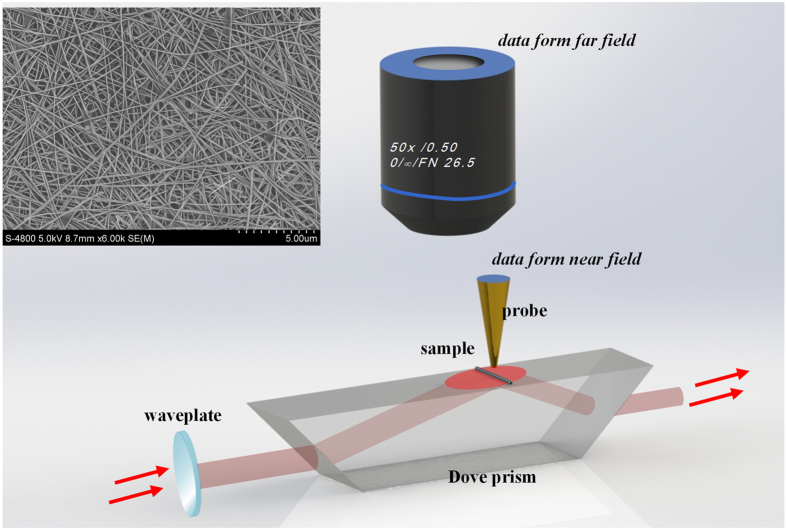
The schematic of experimental setups. Inserted is the SEM photograph of chemically synthesized Ag nanowires.

The schematic of the experimental setup is shown in Fig. 1. The incident light with wavelength of 632.8 nm,passing through a polarizer for polarization controlling, goes into the Dove prism. The choice of this wavelength is because Ag has relatively lowpropagation loss at 632.8 nm^36^, and the light of this wavelength is commonly used for surface enhanced Raman scattering (SERS)when excited remotely with Ag nanowires^37^,^38^. The evanescent wave is then generated on the upper surface of the Dove prism due to the internal total reflection. The whole nanowire is simultaneously excited by the evanescent wave, because of the incident light spot size on the prism surface much larger than the nanowire length. The optical microscope (OM) and the scanning near-filed optical microscope (SNOM) are combined for far-and near-field detection. A super long working distance objective lens(50X, N.A. = 0.5)is used to collect the far field signal while a tapered fiber tip coated with gold film (Super Sensor) is used to measure the near-field one. The diameter of the hole on the tip is measured to be ~200 nm, which means that resolution of the SNOM is ~200 nm (higher than the diffraction limit of 316.4 nm because it is mainly determined by the hole size when the tip is close to the sample surface.

### Perpendicular Situation

**Figure 2:**
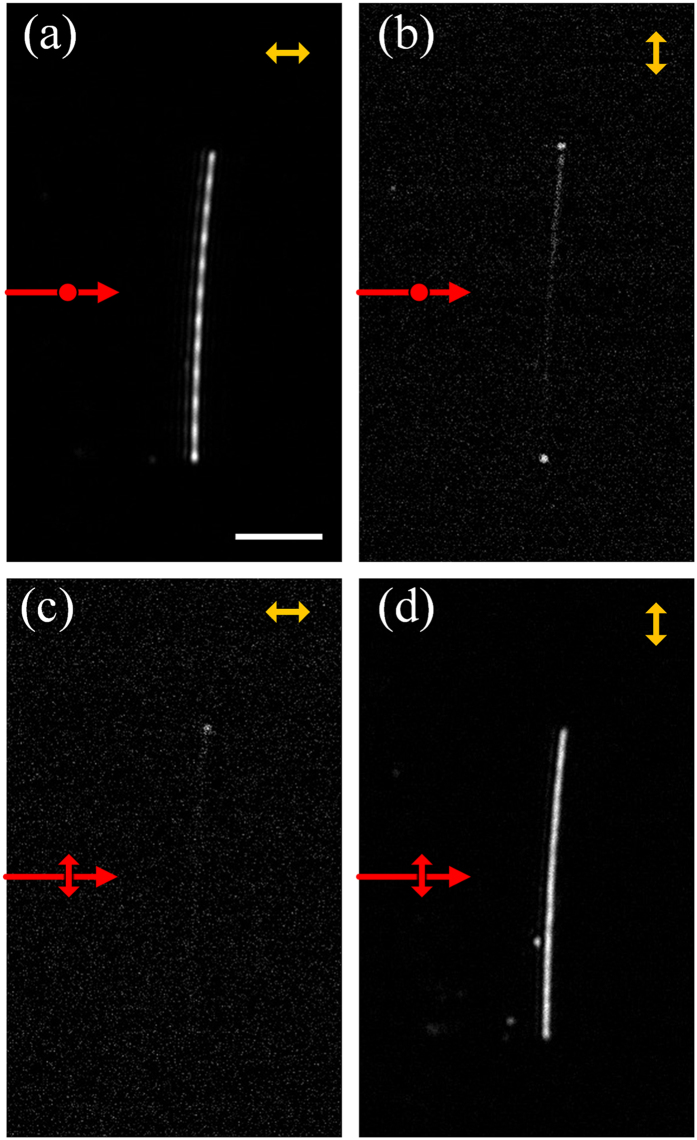
Polarization images of the Ag nanowire. Yellow arrows indicate the polarization direction of the analyzer and red arrows indicate the wave vector and polarization state of the incident light. (**a**,**b**) are *p*- and *s*-polarized images when the nanowire is excited by the *p*-polarized light. (**c**,**d**)are *p*- and *s*-polarized images when the nanowire is excited by the *s*-polarized light. The scale bar in (**a**) is 10 μm.

The polarization property of the single Ag nanowire was firstly investigated when the nanowire axis is perpendicular to the incident plane of light (Fig. 1).The length and diameter of this nanowire is 37.6 μm and ~300 nm, respectively. Typical images of the nanowire observed by the OM in two different polarizations are shown in Fig. 2.

In the condition of the *p*-polarized excitation, the observed intensity distribution of the nanowire is markedly different when the polarization direction of analyzer is changed. When the polarization direction of analyzer (the yellow arrow) is consistence with that of incident light, periodic pattern with average period of 2.99 μm can be clearly seen, as shown in Fig. 2a, implying that the radiating light of the period pattern is in the *p*-polarized state. When the polarization direction of the analyzer is perpendicular to that of incident light, two bright spots can clearly be observed from two ends of the nanowire while the rest of the nanowire is invisible, as given in Fig. 2b.

In the case of the *s*-polarized excitation, the light polarization state of the nanowire is also detected when the polarization direction of analyzer is changed. As the polarization direction of the analyzer is perpendicular to that of the incident light, only two ends of the nanowire give out light while the rest of the nanowire is lightless, as shown in Fig. 2c. By keeping consistence of the analyzer polarization direction and the incident light, the whole nanowire structure is bright and no obvious optical pattern can be observed, in Fig. 2d. Above results shown in Fig. 2 reveal that the light from the nanowire is strongly polarization-dependent while the light from two ends of the nanowire is insensitive to polarization.

**Figure 3:**
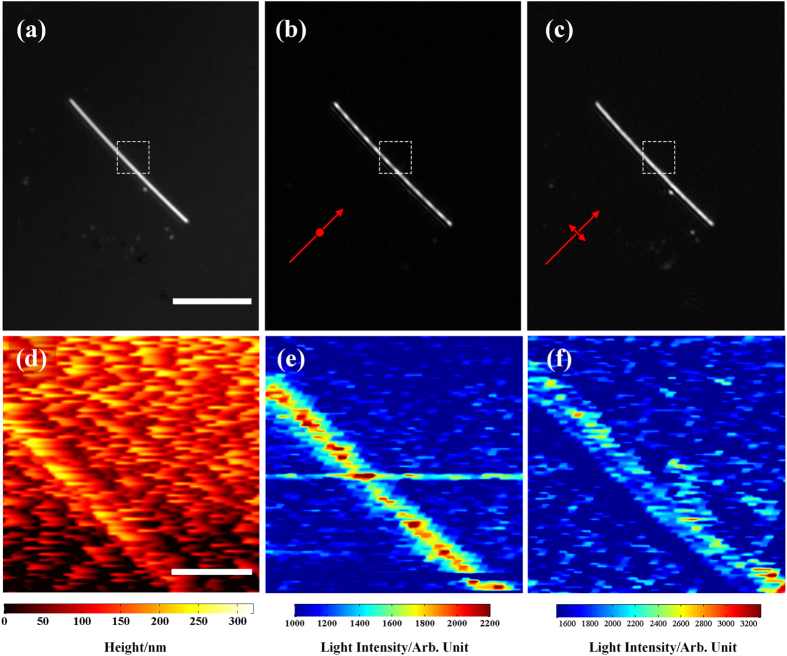
(**a**) Reflected image of the Ag nanowire. (**b**,**c**) Dark field images of the nanowire excited by the *p*-and *s*-polarized light, respectively. The scale bar in (**a**) is 16 μm. (**d**) Topographic image of a section of the nanowire,where the scale bar is 2 μm. (**e**,**f**) Near-field light intensity distribution of the nanowire, captured in the selected area marked by the white dash box in (**a**).

In order to explore the origin of the periodic pattern, far- and near-field measurements of the same Ag nanowire were performed. Figure 3a–c show three images taken in the reflection mode, *p*-polarized and *s*-polarized excitation without the analyzer, respectively. As the previous results shown in Fig. 2, the period pattern can be clearly seen in Fig. 3b. The near-field images were then captured in the selected area marked by the white dashed box in Fig. 3a–c with the scan size of 7 × 7 μm^2^. Figure 3d shows the topographic image of the nanowire, from which the diameter of the nanowire was reconfirmed to be 300 nm. Figure 3e shows the near-field image of the nanowire illuminated by the *p*-polarized light and periodic pattern can also be seen in the near-field region. The average period is 0.98 μm, which is 1/3 of the period measured in the far field region in Fig. 3b. When the excitation light is in *s*-polarized state, no obvious periodic pattern can be resolved in the near-field region, as shown in Fig. 3f.

### Parallel Situation

**Figure 4:**
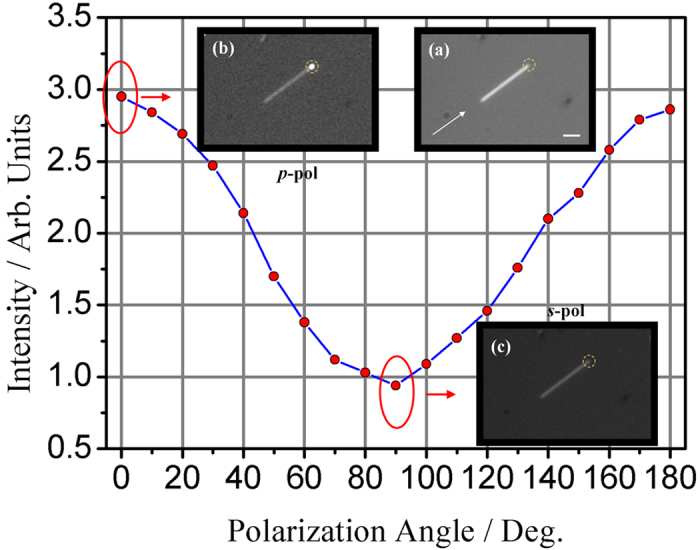
Light intensity against the polarizationstate of incident light. (**a**) the bright field image of the Ag nanowires. The white arrow in (**a**) indicates the wave vectors of the incident light. (**b,c**) the scattering image of the Ag nanowires excited by *p*- and*s*-polarized light, respectively. In order to show the position of the nanowire, the white light is also on in (**b,c**). Scale bar in (**a**) is 2.5 μm.

Propagation property of another single Ag nanowire was studied when the nanowire was positioned in the incident plane by the micromanipulator devices. The Ag nanowire was excited by the evanescent field produced on the up surface of the dove prism. Optical images of the Ag nanowire can be seen through the OM and light intensity value can be read out from a selected area in the CCD equipped on the OM. In the experiment, the circle area of 2 μm in diameter around the output end of the nanowire is selected and shown in Fig. 4(a). When the polarization angle of the incident light was changed, light intensity from the output end was recorded simultaneously, giving relationship between light intensity from the output end and incident polarization angle. Figure 4a shows the reflected image of the nanowire with the length of 9.78 μm. The wave vector of the incident light is represented by the white arrow. The light intensity from the distal end of the nanowire was measured. It is found that the light intensity changes periodically with incident polarization directions. When the polarization angle is at 0° with respect to *p*-polarization (*E*-field component of the excitation light in the incident plane), the collected light intensity has a maximum value, and the distal end of the nanowire is bright, as shown in Fig. 4b. While the polarization angle is at 90°, corresponding to *s*-polarization(*E*-field component perpendicular to the incident plane), the collected light intensity has a minimum value, and the distal end of the nanowire becomes dim, as shown in Fig. 4c.The intensity curve as a function of the polarization angle is given in Fig. 4, showing that the light intensity has a strong polarization dependence of incident light.

**Figure 5:**
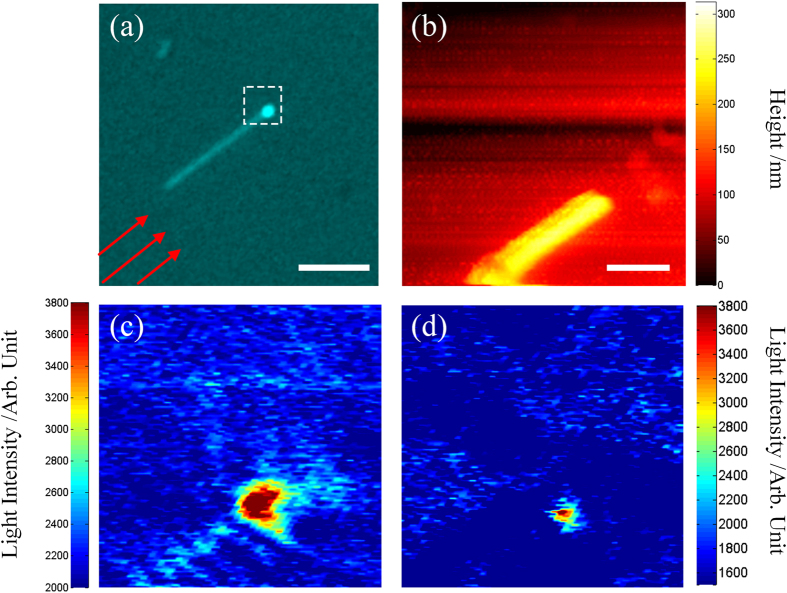
(**a**) Bright field image of the Ag nanowire excited by the *p*-polarized incident light. Scale bar is 5 μm. (**b**) Topography image of the exit end of the Ag nanowire. (**c**,**d**) Near-field light intensity distribution of the Ag nanowire when it is excited by the *p*-and *s*-polarized light respectively. Scale bar is (**b**) is 500 nm.

The near-field measurements at the region around the distal end of the nanowire were performed for more details. As shown in Fig. 5a, the selected area of 2.5 × 2.5 μm^2^ is marked by the white box and the wave vector of the incident light is indicated by red arrows. Figure 5b gives a typical topographic image of the nanowire. Corresponding near-field optical images excited by the *p*- and *s*-polarized light are shown in Fig. 5c,d, respectively. In Fig. 5c, periodic pattern along the nanowire is clearly observed and the average period is measured to be 196.5 nm. A bright spot appears at the distal end of the nanowire in Fig. 5c, while only a smaller bright spot remains at the same position in Fig. 5d.

This periodic pattern results from standing wave due to SPP interference, which will be discussed below. Here, it should be mentioned that the phase change reflected by the end face is of importance for plasmonic waveguides, which can be retrieved from the pattern in Fig. 5 cand based on the equation *ϕ* = (π−*k*_SPP_*l*),where *k*_*SPP*_ is wave number of SPP and *l* is the distance between the first standing wave anti node and the nanowire end face. In the present experiment, the phase change of SPP in the end face is estimated to be 61.7°. Thus, the near field optical image provides a direct method to obtain the phase change comparing to the reported method^39^.

## Discussion

When the nanowire is placed perpendicular to the incident plane, the light intensity (Fig. 2) measured by the far-field optical microscope equipped CCD involves the scattering and absorption (i.e. extinction). As the nanowire is excited by the evanescent wave, the scattered light is collected by the objective lens with a maximum angle of 60°.The scattering and absorption of evanescent waves by small spherical particles has been studied in the framework of generalized Mie theory^40^,^41^,^42^. It was reported that the scattering cross-sections of spherical particles is larger for *p*-polarized evanescent waves than for *s*-polarized ones. A polarization dependence of scattering and extinction of evanescent waves by spherical particles was shown. It was pointed out that contributions of higher multipoles are greatly enhanced in the scattering spectra due to the lager gradient of the intensity of evanescent wave perpendicular to the surface. In particularly, the enhancements of higher multipoles get more obvious when surface plasmon resonances of metallic particles are excited by *p*-polarized evanescent wave. For example, enhanced higher multipoles of Ag particles with a diameter of 400 nm were observed in this case, which make a contribution to the visible spectra. In addition, the presence of the substrate surface also changes the angular intensity distribution in the far-field due to the effect of multiple scattering at surface. This effect becomes strongest when the particle is in contact with the surface^43^.

Such properties of spherical particles can also be sustained by metal structures of cylinder-like geometry with cross section sizes smaller than the wavelength of the exciting light and a quasi-infinite extension along the symmetry axis, i.e. nanowires. The significant difference between them is that spherical geometry allows polarization independent plasmon excitation, while plasmon resonances in nanowires can only be excited by an electric field oriented perpendicularly to the wire axis, because only this direction is a spatial confinement of the electrons provided by the interface of the nanowire and the adjacent medium. In brief the strength of scattering depends on the polarization of the incident wave, plasmon resonances and the presence of the substrate.

Based on the discussion above, the results in Fig. 2 can simply be explained. Owing to strong scattering, the nanowire on the glass substrate is able to convert partially the evanescent to radiative waves, which is detectable in the far field. By comparing Fig. 2a,c with Fig. 2b,d, it is evident that the light scattering of the evanescent wave depends strongly on the polarization direction of the incident light. In the case the nanowire could be regarded as a linear polarizer or a light source, which can be turned on and off with changing the polarization direction of the incident light.

Surface plasmon resonances can be excited in the case of *p*-polarized evanescent wave. However, due to the limitation of the present experiment setup, we cannot measure the scattering spectra and discuss the contribution of surface plasmon resonances to the scattering. From the reported resultit is known that the surface plasmon resonances in the visible light band can be excited on the Ag nanowire of 300 nm in diameter^44^. If this result is valid for the present measurement, the scattering light should be influenced by the surface plasmon resonance.

It should be mentioned that the patterns observed both in the far-field (Fig. 2a) and near-field (Fig. 3e) are interesting phenomena. To understand the origins of the periodic pattern, the oscillating dipole model is considered^45^. Instead of a single dipole, a chain of dipoles placed one by one on the glass/air interface is used to simulate the case (see Figure S1 in the Supplementary Information). Under the *p*-polarized excitation, we consider a series of dipoles oscillating in the substrate surface. Their orientation is parallel with each other and these dipoles oscillate with the same frequency and phase. The construction and deconstruction of light from the each dipole source forms the period pattern (see Figure S1b in the Supplementary Information). Under the *s*-polarized excitation, as the incident polarization state is along the nanowire, we consider another case that a series of dipoles is placed end to end. No obvious period pattern can be seen under this configuration. Although the exact period patterns depend on the geometric parameters, there is a striking similarity between the simulated periodic patterns and the experimental ones shown in Figs 2a and 3e. This model helps us to explain the situation in the Ag nanowire. Under the *p*-polarized light, the *E*-field component oscillates in the incident plane. At this case, the cross section of the nanowire can be seen a nanosphere, and surface plasmon mode can be excited on the perpendicular direction. This is one origins for the dipole source.

The difference between the pattern periods observed in the near-field and far-field is due to the difference between the resolutions of the SNOM and the OM. As mentioned previously, the resolution of the SNOM is ~200 nm and that of the optical microscope is ideally 770 nm for the illumination light of ~632.8 nm and NA of 0.5. Obviously, the SNOM with higher resolution is able to provide finer and richer optical information. One of the important issues we have to do would focus on exploiting the complementary information among the two different methods to further study the optical properties of the nanowires excited by evanescent waves. In addition, polarization-independence of the light from the distal end might be due to the curved geometric boundary and the surface plasmon resonances, which would make the scattered light insensitive to polarization^46^.

**Figure 6:**
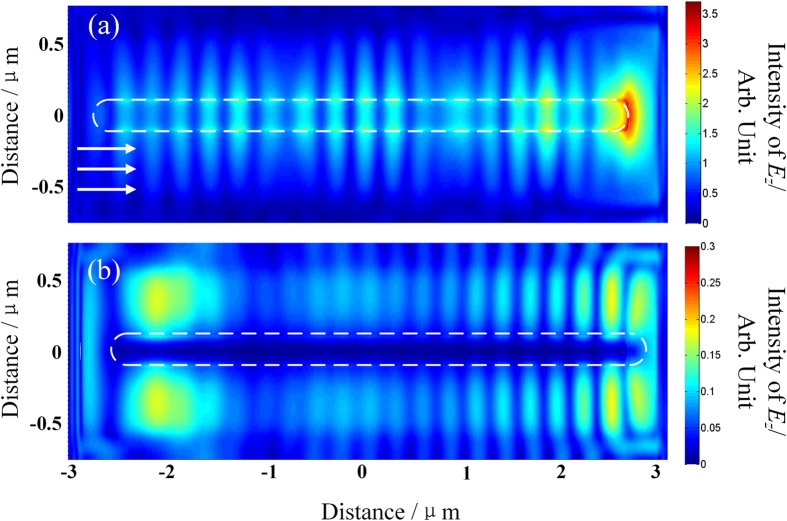
(**a,b**) Calculated near-field *E*_*z*_ distribution on the nanowire when it is excited by the *p*-and *s*-polarized light, respectively. White arrows indicate the wave vector of the incident light.

When the orientation of nanowire is moved parallel to the incident plane, light is just scattered by the distal end. Periodic pattern along the nanowire can be resolved in the near-field region, which can be explained as SPP excitation. It is well known that SPP modes at the metal/insulator interface can only be excited by the TM polarization light (*p*-polarization)^47^, and the wavelength of SPP is smaller than that of the excitation light^48^. When the nanowire is placed in the evanescent wave produced by the *p*-polarized light, SPP is excited. The forward SPP wave and the backward one reflected by the distal end interfere with each other, forming the standing wave(Fig. 5c). We also performed near-field calculation of *E*_*z*_ intensity in *XY* plane using finite difference time domain (FDTD) method. The configuration in the simulation is the same as that in the experiment. Figure 6a,b show the calculated results when the nanowire is excited by the *p*- and *s*-polarized light, respectively. The calculated results confirm that only *p*-polarized light can excite the periodic pattern on the nanowire. Furthermore, from Fig. 5c, the wavelength of the interference pattern is estimated to be 393 nm, smaller than that of the incident light (633 nm). These results above indicate that the period pattern originates from SPP excitation.

**Figure 7:**
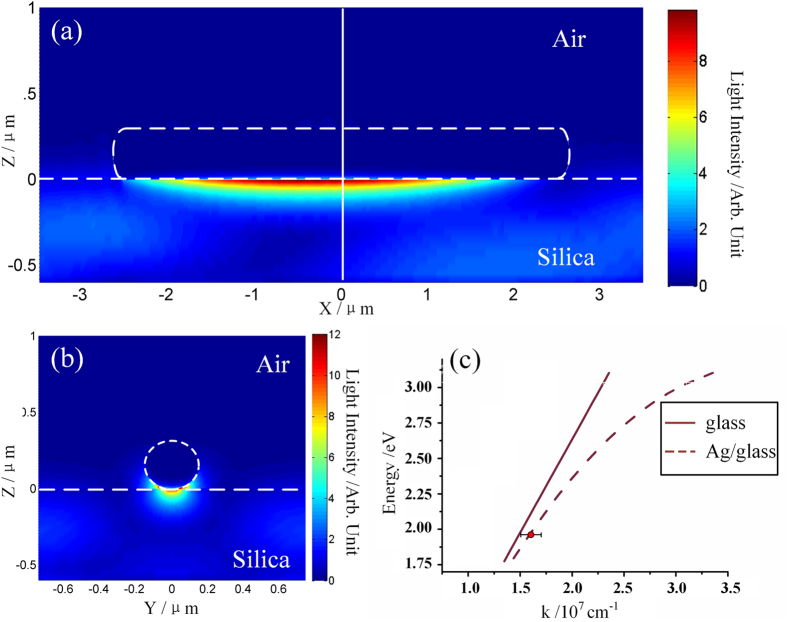
(**a,b**) Calculated *E*-field intensity distribution of the Ag nanowire in *XZ* plane and *YZ* plane respectively. The white solid line in (**a**) indicates sectional position of (**b**). The nanowire is excited by the *p*-polarized light. (**c**) Dispersion relationship of photons in the glass (black solid) and SPP on the Ag/glass interface (black dashed). Red circle represents the SPP propagation mode retrieved from the experiment.

The propagation mode of SPP is also analyzed. Figure 7c shows the theoretical dispersion curve of SPP propagation mode along the interface of Ag/glass. The wavelength of SPP has been measured from Fig. 5c and thus the “dispersion data” of the Ag nanowire/glass substrate can be obtained (red dot in Fig. 7c). It is found that the dispersion data is just located on the dispersion curve of Ag/glass interface, indicating that the electromagnetic energy is transported between the Ag nanowire and the glass substrate. The distribution of light intensity in *XZ* and *YZ* plane was also simulated by FDTD calculation. The results are given in Fig. 7a,b, where Fig. 7a shows light intensity distribution along the nanowire in the *XZ* plane, and Fig. 7b is the distribution of the cross section in *YZ* plane, marked by the white vertical dashed lines in Fig. 7a. The simulation results show that most of the energy concentrates on the interface of Ag nanowire and glass substrate, further supporting our experimental results.

When SPP is excited by the evanescent wave in the *p*-polarized light, it propagates along the nanowire/glass interface and scatters/emits at the distal end. The light intensity at the distal end results from the direct scattering of the evanescent wave as well as SPP emission. In the *s*-polarized situation, however, SPP can hardly be excited and the collected intensity is only from the scattering of evanescent wave by the nanowire end. Therefore, polarization-dependent light intensity of the distal end is due to the SPP excitation. Near-field images are able to provide more information including SPP interference pattern. Key physical quantities can be derived from the high-resolution SNOM images. Combination of the near- and far field methods will provide complementary information for further understanding of these effects.

In previously published literatures, propagation mode of a supported Ag nanowire on the glass substrate has been reported^48^. It is found that the dispersion curve of the nanowire is located on the right side of the dispersion curve of the air, glass and air/glass substrate. In our experiment we found that the dispersion mode of nanowire is located on the Ag/glass interface. As the diameter of the Ag nanowire used in our experiment is 300 nm, surface plasmon modes on the up and bottom surface of the nanowire have less opportunity of couple with each other, because the diameter of the nanowire is smaller compared with that in the previous literature. The propagation mode measured in our system is decoupled.

In summary, the single Ag nanowire/glass substrate system excited by the evanescent wave demonstrates completely different properties, depending on its orientation to the incident plane and polarization direction of light. When placing the nanowire perpendicular to the incident plane, it works as a scatter to convert partially the evanescent to radiate waves. The intensity of scattering light depends strongly on the polarization of the incident light and can be turned with changing the polarization direction. If the nanowire is positioned along the incident plane, it operates as a waveguide for SPP propagation. Near-field and simulation results reveal that *p*-polarized excitation plays an important role in surface plasmons generation. The present setup can provide a wealth of information on the nanowire’s optical properties. Future work will focus on the measurement of the far-/near-field scattering and surface plasmon resonance spectra of one-dimensional metal materials.

## Methods

The Ag nanowires are synthesized by the liquid growth method^49^,^50^,^51^. A typical synthesis progress is as follows. First of all, 0.05094 g AgNO3 is dissolved into 3 ml Ethylene Glycol (EG) and 0.1998 g poly(vinylpyrrolidone) (PVP) is dissolved into another 3 ml EG solution respectively. The PVP/EG solution is stirred preheated at 70 °C for 40 minutes to ensure the PVP is dissolved fully. Meanwhile another 5 mL EG is preheated in an oil bath pot at 160 °C for 40 minutes. Next the AgNO_3_/EG and PVP/EG solution is dropped into the preheated 5 ml EG solution at the same time and the dropping rate should be controlled at 0.3 ml per minute. After the dropping process is finished, the mixture solution is stirred and react at 160 °C for 1 hour. The stir rate is controlled at no more than 260 rpm. When the color of reaction solution becomes light gray it indicates Ag nanowires has been produced. At last the reaction solution is centrifuged and rinsed (inserted SEM image in Fig. 1).A droplet of 2 μL Ag nanowire solution is dripped onto the surface of a Dove prism and is left to dry in the ambient condition, yielding well separated individual nanowires.

The optical microscope used here is Olympus microscope, and the collecting objective lens is the Olympus super long distance objective lens (N.A. = 0.5, W.D. = 10.6, 50X). The near-field tip (Super Sensor^TM^) is a tapered fiber tip coated with gold film, and the hole on the tip is manufactured is 200 nm. The feedback of SNOM system is the tapping mode feedback, and the sampling rate is 20 ms/point. The Dove prism (GCL-030602) is from Daheng Optics Cooperation, the length of the up surface and the height is 42.28 cm and 10 cm, respectively. The micromanipulator is MMO-202ND from Narishige Group Company.

In the FDTD model, the Ag nanowire is built as a cylinder capped with a hemisphere at each end. The length and the diameter of the wire is 5.3 and 0.3 μm, respectively. And the permittivity of Ag is taken from the data of Johnson and Christy in 1972. For the FDTD calculation parameters, the minimum cell size and the time step is set as 2 nm and 0.075 fs respectively. Besides, the boundary condition (BC) is set as perfectly matching layer (PML) at the up and bottom surface of calculation region, periodic BC at the front and back surface, and Bloch BC at the left and right surface, which corresponds to the input and output surface of light source.

## Additional Information

**How to cite this article**: Yang, M. *et al*. Orientation-and polarization-dependent optical properties of the single Ag nanowire/glass substrate system excited by the evanescent wave. *Sci. Rep*. **6**, 25633; doi: 10.1038/srep25633 (2016).

## Supplementary Material

Supplementary Information
