# Supplementary material for: Orientation-and polarization-dependent optical properties of the single Ag nanowire/glass substrate system excited by the evanescent wave
Source: Sci Rep. 2016 May 9;6:25633. doi: 10.1038/srep25633 (PMC4860604; doi:10.1038/srep25633)
Supplement: Supplementary Information [file srep25633-s1.docx]

Supplementary Information for

Orientation- and polarization-dependent optical properties of the single Ag nanowire/glass substrate system excited by the evanescent wave

by Mu Yang, Wei Cai, Yingjie Wang, Mengtao Sun &Guangyi Shang

**S1.A chain of dipoles model**

A series of separated dipole source are used to simulate the near-field optical distribution on the nanowire under different polarization excitation[^1^](#_ENREF_1).This dipole sources has the same frequency and phase. The frequency is in agreement with the incident light of 632.8 nm. For the *p*-polarized case, 8 dipole is placed parallel with each other on the glass substrate. The distance between the dipoles and the substrate is set as 10 nm. And the interval between each dipole is set as 100 nm. Figure S1(a) shows a schematic of the parallel dipoles in top view and Figure S1(b) is the simulation results by FDTD software. An obvious period pattern is repeated by the simulation results. For the *s*-polarized case, as the incident polarization state changes, the orientation of the dipole also changes for 90º. Moreover, we use more dipole (29) to mimic the *s*-polarized cases. The number is the dipole in Figure S1(c) is not proportional. The interval between the dipole are also decreased (25 nm) due to the good conductivity of the silver. No obvious period pattern on the quasi-continuous dipole source situation.


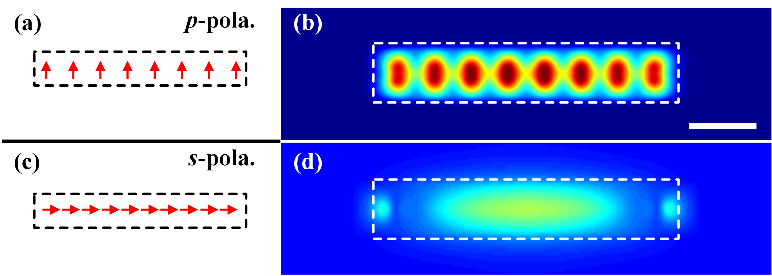


**Figure S1 |**The simulation results by the chain of dipoles mode. (a) and (b) are the schematic of the dipoles chain parallel with each other and the simulated periodic pattern in the top view, respectively. (c) and (d) are the schematic of the dipoles chain end to end and the simulated results, respectively.

1. Timur, S. *et al.* Unidirectional broadband light emission from supported plasmonic nanowires. *Nano. Lett.***11**, 706-711 (2011).
